# A Study of the Effects of Latent Iron Deficiency on Measures of Cognition: A Pilot Randomised Controlled Trial of Iron Supplementation in Young Women 

**DOI:** 10.3390/nu6062419

**Published:** 2014-06-23

**Authors:** Alecia J. Leonard, Kerry A. Chalmers, Clare E. Collins, Amanda J. Patterson

**Affiliations:** 1 Priority Research Centre in Physical Activity and Nutrition and School of Health Sciences, Faculty of Health and Medicine, University of Newcastle, Callaghan, NSW 2308, Australia; E-Mails: Alecia.Leonard@uon.ed.au (A.J.L.); Clare.Collins@newcastle.edu.au (C.E.C.); Amanda.Patterson@newcastle.edu.au (A.J.P.); 2 School of Psychology, Faculty of Science and IT, University of Newcastle, Callaghan, NSW 2308, Australia

**Keywords:** iron, women, cognition, memory, attention, supplementation

## Abstract

Rates of iron deficiency are high amongst healthy young women. Cognitive impairment occurs secondary to iron deficiency in infants and children, but evaluation of the impact on cognition among young women is inconsistent. The aim was to determine the suitability of the IntegNeuro test battery for assessing cognitive function in iron-deficient and iron-sufficient young women. A pilot double-blinded, placebo-controlled intervention trial was conducted in iron-deficient (serum ferritin ≤ 20 μg/L and haemoglobin > 120 g/L) and iron-sufficient young women (18–35 years). Cognitive function and haematological markers of iron status were measured at baseline and follow-up. Iron-deficient participants (*n* = 24) were randomised to receive placebo, 60 mg or 80 mg elemental iron daily supplements for 16 weeks. A control group of iron-sufficient participants (*n* = 8) was allocated to placebo. Change scores for Impulsivity and Attention were significantly greater in plasma ferritin improvers than in non-improvers (*p* = 0.004, *p* = 0.026). IntegNeuro was easy to administer and acceptable to young women. Based on the differences in Memory and Attention scores between iron-deficient participants on iron treatment and those on placebo, it was decided that between 26 and 84 participants would be required in each iron treatment group for an adequately powered extension of this pilot RCT.

## 1. Introduction

Iron deficiency is the most prevalent nutritional deficiency worldwide [1]. No population group is unaffected, but rates are highest for young women, infants and children in their first two years of life [2]. Young women are at particular risk of iron deficiency due to increased iron requirements secondary to menstruation and pregnancy. Up to two thirds of young women in developing countries suffer from iron deficiency [3]. However, it is not only a phenomenon of developing nations, with prevalence rates of between 10% and 20% found in the U.S. and Europe [2].

Latent iron deficiency is characterised by individuals having serum ferritin ≤ 20 μg/L and haemoglobin > 120 g/L [2]. Iron-deficiency anaemia is the most severe form of iron deficiency and is characterised by having haemoglobin ≤120 g/L, in addition to having low serum ferritin [4].

There is now good evidence of an association between iron deficiency and impaired cognitive function in infants and children [5,6,7,8,9]. Cognition is important for quality of life and encompasses various functions including memory, attention and concentration [4]. The exact mechanism by which iron deficiency affects the brain is not well understood. Possibilities include abnormalities in neurotransmitter metabolism, decreased myelin formation, and alterations in brain energy metabolism [10,11].

Studies in infants and children have shown that iron deficiency without anaemia can cause changes in brain development and function [12]. These changes have been shown to specifically affect concentration, attention and short-term memory [5,7,8,13]. A review of longitudinal studies found that adolescents who had experienced iron-deficiency anaemia during infancy continued to perform less well in spatial memory and selective attention tasks compared to peers who had adequate iron status in infancy [14].

Due to the high rates of iron deficiency in young women, it is important to conduct well designed randomised controlled trials to investigate whether iron deficiency is associated with deficits in cognitive function in this population. Of the studies that have been conducted in this population, some have found poorer cognitive performance in iron-deficient participants compared with iron-sufficient participants [15,16,17], whereas others have found no difference [11,18,19]. Following iron supplementation, the majority of studies have reported an improvement in cognitive function in young women who were iron-deficient at baseline [11,15,16,17,18,19,20,21]. However, some studies have found no difference [22]. Two systematic reviews have examined this topic and concluded that there is some evidence of an effect but findings are confounded by extremely varied methodologies, particularly with regard to measures of cognitive function and definitions of iron deficiency [4,23].

The most recent work in this area has been conducted by Murray-Kolb and Beard (2007) [16] and Blanton (2013) [24]. Both conducted prospective randomised controlled intervention trials in young women (18–35 years and 18–30 years, respectively) of varied iron status. Murray-Kolb and Beard used iron supplements and Blanton used high bioavailable iron meals as the intervention. Murray-Kolb and Beard found that at baseline, iron-sufficient women performed better on cognitive tasks and completed them faster than women with iron deficiency anaemia (haemoglobin < 120 g/L). They also showed that participants with latent iron deficiency (haemoglobin 105–119 g/L and ≥2 abnormal iron status values) performed intermediary between the two extremes of iron status which compared the 1st *vs.* 5th quintiles. There were no significant differences between latent iron-deficient and iron-sufficient participants. After treatment with iron supplements, a significant improvement in serum ferritin was associated with improvements in cognitive performance, while improvement in haemoglobin, defined as percentage change greater than 4.4%, was related to improved speed in completing the cognitive tasks [16]. Similarly, in a trial comparing beef and non-beef lunches three times weekly for 16 weeks, Blanton [24] reported that participants whose ferritin increased had significantly greater improvements in planning, speed, spatial working memory and strategy than those whose ferritin did not increase.

In addition to using different interventions, the studies also differed with regard to the cognitive tests used. Murray-Kolb and Beard [16] used Detterman’s Cognitive Abilities Test whereas Blanton [24] used the Cambridge Neuropsychological Test Automated Battery. Both batteries claim to measure similar domains, including verbal memory, spatial memory and visual information processing. However, they include different individual cognitive tests and are therefore not directly comparable. The two systematic reviews mentioned above found that some studies have used individual tests such as Digit Span or Block Design [23], whereas others have used composite test batteries such as the Wechsler Memory Scale-Revised (WMS-R), Wechsler Adult Intelligence Scale-Revised (WAIS-R), and Hopkins Verbal Learning Test [4,23].

In order to achieve some homogeneity in the area of iron deficiency and cognition in adults, research groups need to use the same tools in studies of similar design. The preferred tool needs to have good reliability and validity and standardised administration procedures. IntegNeuro is a battery of cognitive tests that has good reliability and validity. Paul *et al.* [25] reported on the validity of the IntegNeuro battery in assessing seven cognitive domains (Memory, Response Speed, Impulsivity, Attention, Information Processing, Executive Function, Emotion Identification) in a sample of 50 healthy adults (25 women and 25 men, age 22–80 years). This study assessed validity, conducting correlation analyses between IntegNeuro and paper based tests, and examined the influence of age, education and sex on test results. They found strong relationships between IntegNeuro tests and standard measures of cognitive function [25]. Clark *et al*. [26] examined the effects of age, gender and education on cognitive function using the IntegNeuro battery and reported its sensitivity regarding the assessment of cognition.

The current research examines the effect of latent iron deficiency on cognitive function in young women. The aim was to determine the suitability of the IntegNeuro battery for assessing cognitive function in iron-deficient and iron-sufficient women in a double-blind placebo-controlled trial.

## 2. Experimental Section

This study was conducted at the University of Newcastle, Callaghan Campus in NSW, Australia, between April 2010 and April 2013. Ethics approval was provided by the Human Research Ethics Committee at University of Newcastle (H-2010-1079). Women aged 18–35 years were recruited via flyers, promotion in lectures, through the Hunter Medical Research Institute volunteer register and by word-of-mouth. All interested individuals were screened for eligibility against inclusion criteria. These were: female, 18–35 years; BMI 18–30 kg/m^2^; English as primary language; not iron deficient within the last 12 months; not currently taking iron supplementation (those on a standard multivitamin were eligible to participate); no chronic medical condition; not taking medication that could potentially interfere with results (anti-inflammatory medications, antacids, histamine receptor antagonists, proton pump inhibitor); ability to provide blood samples for biomarkers of iron status; not pregnant, or planning a pregnancy within the following 4 months; available to participate in the intervention for 4 months. Those eligible were provided with an information statement and informed consent was obtained prior to the commencement of the study.

**Figure 1:**
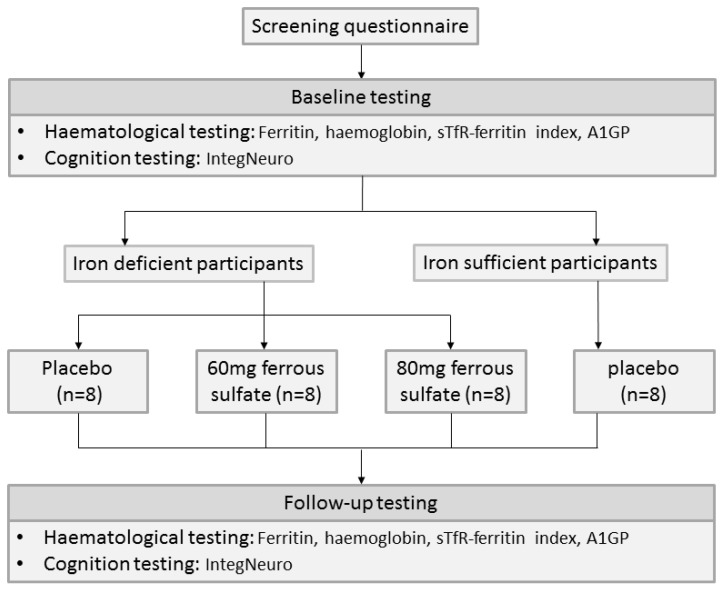
Flow chart describing the participant flow through a pilot double-blinded, placebo-controlled randomised control trial of the effect of iron supplementation (60 mg or 80 mg iron or placebo for 16 weeks) on the cognitive function of iron-deficient (ferritin < 20 μg/L, haemoglobin ≥ 120 g/L) and iron-sufficient (ferritin ≥ 20 μg/L, haemoglobin ≥ 120 g/L) women (18–35 years).

The study included baseline cognitive and haematological testing, a 16 week intervention with two separate doses of elemental iron (60 mg or 80 mg) in the form of ferrous sulfate or placebo, an iron-sufficient control group allocated to placebo, and follow-up testing. Figure 1 shows the recruitment process and study flow chart.

Ferritin = serum ferritin; sTfR-ferritin index = soluble transferrin receptor to serum ferritin ratio; A1GP = alpha-1-glycoprotein; IntegNeuro = a self-administered (touch screen) battery for assessing cognitive function including the domains of Memory, Response Speed, Impulsivity, Attention, Information Processing, Executive Function, Emotion Identification.

### 2.1. Haematological Testing

Serum ferritin, haemoglobin, soluble transferrin receptor (sTfR) and alpha-1-glycoprotein (an inflammatory marker) were measured at baseline and follow-up. Blood samples were collected by Hunter Area Pathology Service. Latent iron deficiency was defined as having ferritin < 20 µg/L and all other markers within reference ranges (haemoglobin 115–165 g/L [27], sTfR 0.9–2.30 mg/L [28,29], alpha-1 glycoprotein 0.51–1.17 g/L [30]). sTfR is expected to be raised in the presence of iron deficiency. However, all participants in the current study had normal sTfR. Ferritin reflects storage iron and sTfR reflects functional iron, therefore, sTfR-Ferritin Index (sTfR-Index) has been proposed as a measure that may increase the accuracy of iron deficiency diagnosis [31]. Anaemic participants (ferritin < 20 µg/L, haemoglobin < 120g/L) were excluded from the remainder of the trial and data analyses. Participants were classified as ferritin improvers or non-improvers according to the criteria used by Blanton [24] and Murray-Kolb and Beard [16]. This criterion considers whether participants had a change in ferritin greater or less than the known biological day-to-day variation (27%) [32,33,34]. Participants’ percentage ferritin change during the trial was calculated. Participants with percentage changes above 27% were deemed ferritin improvers and those with percentage changes of ≤ 27% were considered ferritin non-improvers. Participants were also classified as haemoglobin improvers or non-improvers based on whether they had a change in haemoglobin greater or less than the known biological day-to-day variation (4.4%) [16,24].

### 2.2. Cognitive Testing

Cognitive function was measured at baseline and follow-up using the IntegNeuro Battery of Cognitive Tests developed by the Brain Resource Company [35]. The IntegNeuro battery includes the following cognitive domains, with the individual tasks within each domain in parentheses: Memory (recall, recognition, digit span, span of visual memory); Response Speed (motor tapping); Impulsivity (reaction time); Attention (sustained attention); Information Processing (switching of attention, choice reaction time, verbal interference); Executive Function (maze) and Emotion Identification (emotion recognition).

The IntegNeuro battery was self-administered using a touchscreen to present the tasks and record responses. A headset was used to deliver auditory instructions and tasks. The tests yield a standardised score out of ten (sten) for each of the cognitive domains. Sten scores divide the score scale into ten units. Each unit has a bandwidth of half a standard deviation, except the highest Sten score (10) which extends upwards from two standard deviations above the mean, and the lowest Sten score (1), which extends downwards from two standard deviations below the mean [35,36]. Higher Sten scores indicate better cognitive function.

Practice trials were included before scored trials for relevant cognitive tasks. Cognition testing was conducted in a private, sound-proof testing room. The experimenter was present in the adjoining room but there was no communication with participants during testing. Participants completed the IntegNeuro battery in one session of approximately 50 min.

### 2.3. Treatment Randomisation

Participants with latent iron deficiency were randomised to one of two doses of ferrous sulfate (60 mg or 80 mg), or placebo. The first eight iron-sufficient (ferritin ≥ 20 μg/L) participants who were screened were invited into the intervention as a control group. A single blinding approach was used with the control group who all received placebo capsules, whereby researchers knew they were iron-sufficient, but participants remained blinded to both their iron status and their capsule formulation. Subsequent iron-sufficient participants exited the study following baseline testing. Every four weeks participants were contacted and asked to report possible side effects associated with the treatment. The study piloted the two doses of iron treatment (60 mg and 80 mg) to determine an efficacious dose to which participants could remain blinded. Those results form the basis of another paper [37].

There was no specific time of the day that participants were required to take their capsules. Participants were provided with a tips sheet which included information about taking their capsules 2 h apart from any other medications (except the oral contraceptive pill) as a precaution. The tips sheet also included strategies for remembering to take the capsules, such as placing them next to their toothbrush or in their handbag using the small extra capsule container provided.

### 2.4. Treatment Blinding

Compounding chemists were contracted to provide the iron and placebo supplements. To ensure blinding, the active and placebo supplements were identical in appearance, were packaged in identical containers and tasted the same. Participants were provided with 112 capsules (1 capsule per day for 16 weeks). The researchers and participants remained blinded to the treatment protocol and the randomisation code was held by the compounding chemists only to be broken once the final results were collected. Researchers remained blinded to treatment allocations until all participants had completed the study. A University of Newcastle researcher, external to the study, un-blinded participants to their allocated treatment group and forwarded them a letter explaining their treatment group and their iron status at the follow-up blood assessment. At the end of the intervention participants were asked to guess which treatment they were taking.

### 2.5. Required Sample Size for an Adequately Powered RCT

The sample size calculation was estimated using the difference in cognitive change score results between iron-deficient participants on iron treatment and those on placebo in the current trial. More specifically, data used in this calculation were: 0.05 as the type I error probability; power of 80%; the difference in means from baseline to follow-up for Memory and Attention sten scores for iron-deficient participants taking iron treatment and those taking placebo; and within group standard deviation for each of these cognitive domains.

### 2.6. Statistical Analysis

STATA-IC 11 [38] was used for performing statistical analyses. An alpha level of 0.05 was used for statistical significance. Due to the small sample size and low power to detect significant differences, we also report differences at an alpha level of < 0.10 as marginally significant.

Kruskal Wallis tests were used to examine differences between treatment (60 mg and 80 mg) and no-treatment (placebo and control) groups in iron status, cognitive domain scores, and the individual measures that contribute to the cognitive domain scores, at baseline and follow-up. Kruskal Wallis tests were also used to analyse the effect of treatment (60 mg and 80 mg combined *vs.* placebo and control combined) on change scores (baseline to follow-up) for each cognitive domain and for each of the measures that contribute to the domain scores. Two additional sets of analyses were performed on cognitive changes scores: one with ferritin improvers *versus* non-improvers as the independent variable; the other with haemoglobin improvers *versus* non-improvers as the independent variable.

## 3. Results

### 3.1. Participants

Eligibility screening was completed by 134 young women, of whom 128 were eligible and 95 completed the trial. Of the 24 participants who were iron-deficient at baseline, 19 completed the trial (placebo, *n* = 6; 60 mg iron, *n* = 7; 80 mg iron *n* = 6). Six iron-sufficient participants in the control group, completed. Reasons given for withdrawal were: an unrelated illness (*n* = 3) or being too busy (*n* = 3). One participant gave no reason. Of the 25 participants who completed the trial, the majority was Australian (90%), the mean age was 25.7 ± 4.1 years and 12 participants reported taking the oral contraceptive pill (OCP). There was no difference in age or OCP use across the allocated groups. Participants complied adequately with the intervention. The average number of capsules remaining of the 112 provided to participants was 12 ± 12 (9.6%).

### 3.2. Iron Status

There were no significant differences in ferritin, haemoglobin, sTfR-Index or A1GP between the three iron-deficient groups (60 mg, 80 mg and placebo) at baseline. As expected, iron-sufficient controls had significantly higher ferritin and lower sTfR-Index than iron-deficient groups. Following the intervention, a significant difference in ferritin change score was revealed between treatment groups (60 mg and 80 mg) and no treatment groups (iron-sufficient controls and iron-deficient placebo). There was no significant difference in either haemoglobin or sTfR-Index change scores between iron treatment (60 mg and 80 mg) and no treatment groups (iron-sufficient controls and iron-deficient placebo). A1GP results were within the reference range for all participants pre- and post-intervention. Full details regarding iron status of participants pre- and post-intervention are presented elsewhere [37].

### 3.3. Cognitive Scores

Median cognitive domain scores (and interquartile range, IQR) for each group at baseline and follow-up are presented in Table 1. Medians are reported due to non-normal distributions, primarily due to small sample size.

**Table 1:** Median (and interquartile range) for each cognitive domain score from a pilot double-blinded, placebo-controlled randomised control trial of the effects of iron supplementation (60 or 80 mg iron or placebo for 16 weeks) on the cognitive function of iron-deficient (ferritin < 20 μg/L, haemoglobin ≥ 120 g/L) and iron-sufficient (ferritin ≥ 20 μg/L, haemoglobin ≥ 120 g/L) women (18–35 years), at baseline and follow-up by group (60 or 80 mg iron, iron-deficient placebo or iron-sufficient control).

Cognitive domain	60 mg Iron (*n* = 7)		80 mg Iron (*n* = 6)		Placebo (*n* = 6)		Control ( *n* = 6)	
	Baseline	Follow-up	Baseline	Follow-up	Baseline	Follow-up	Baseline	Follow-up
Memory	6.00 (6.0–7.0)	6.50 (6.5–8.0)	4.25 (3.5–5.0)	4.25 (4.0–5.0)	6.75 (5.0–7.5)	6.50 (4.5–7.5)	7.00 (3.0–8.0)	6.75 (5.0–7.5)
Response Speed	4.50 (3.0–6.0)	6.50 (2.0–7.5)	6.00 (5.0–6.5)	4.00 (3.5–4.5)	5.50 (3.0–7.0)	6.50 (2.0–7.0)	6.00 (4.5–6.5)	6.25 (6.0–6.5)
Impulsivity	6.50 (5.0–7.5)	8.50 (4.0–9.0)	4.75 (4.0–8.0)	6.25 (4.5–9.0)	6.00 (4.5–8.5)	6.50 (5.0–8.5)	7.00 (4.5–9.0)	5.00 (3.5–6.5)
Attention	4.50 (2.5–6.5)	5.00 (3.0–7.0)	4.25 (1.0–8.0)	7.50 (3.5–9.5)	7.25 (4.0–7.5)	5.75 (4.0–7.5)	7.50 (6.0–8.0)	7.00 (4.0–8.0)
Information Processing	6.00 (4.5–9.0)	8.00 (6.0–9.0)	4.50 (4.5–6.0)	6.50 (4.5–8.0)	7.00 (5.0–7.5)	6.50 (6.0–7.0)	7.75 (6.5–8.0)	6.50 (6.5–6.5)
Executive Function	8.00 (7.5–9.0)	7.50 (6.5–8.0)	4.50 (3.0–7.0)	7.00 (6.5–7.5)	7.50 (4.5–8.5)	7.50 (7.0–8.0)	7.50 (7.5–8.5)	7.75 (5.0–10.0)
Emotion Identification	6.50 (3.0–7.5)	4.00 (1.5–8.0)	6.00 (5.0–8.0)	5.75 (4.5–7.0)	6.00 (3.0–6.5)	7.00 (4.0–8.0)	4.00 (1.0–6.5)	4.25 (4.0–6.5)

Note: Scores are Sten scores (0–10), higher scores indicate better performance. Sixty milligrams (60 mg) and 80 mg iron groups took ferrous sulfate. Control: iron-sufficient participants taking placebo. Placebo: iron-deficient participants taking placebo. Statistical analyses on differences in cognitive domain scores between baseline and follow-up for each group were not conducted due to small sample sizes.

#### 3.3.1. Baseline Comparison of Iron-deficient Groups (combined) *vs*. Iron-sufficient Group

Analyses comparing iron-sufficient (controls) and iron-deficient participants (60 mg, 80 mg and placebo groups combined) at baseline revealed no significant between-group differences on any of the cognitive domains (Memory (*p* = 0.523); Response Speed (*p* = 0.652); Impulsivity (*p* = 0.655); Attention (*p* = 0.263); Information Processing (*p* = 0.353); Executive Function (*p* = 0.543), or Emotion Identification (*p* = 0.178)).

#### 3.3.2. Baseline Comparison of Iron-Deficient Groups

Analysis of cognitive domain scores at baseline for the three iron-deficient groups (60 mg iron, 80 mg iron, placebo) revealed a significant difference in the Executive Function domain score (*p* = 0.012). *Post hoc* analysis indicated that the 80 mg iron group had lower Executive Function scores than the 60 mg iron group (*p* = 0.006). There was no significant difference in Executive Function scores between placebo and 60 mg (*p* = 0.411) or placebo and 80 mg groups (*p* = 0.182). There was a marginally significant difference in Memory domain scores between the three iron-deficient groups (*p* < 0.053). *Post hoc* analysis showed the 80 mg iron group had marginally lower Memory scores than the 60 mg group (*p* = 0.098). There was no difference in Memory score between placebo and 60 mg (*p* = 1.000) or placebo and 80 mg groups (*p* = 0.128).

There were no significant differences between the three iron-deficient groups at baseline for Response Speed (*p* = 0.390); Impulsivity (*p* = 0.702); Attention (*p* = 0.537); Information Processing (*p* = 0.272), or Emotion Identification (*p* = 0.767).

#### 3.3.3. Cognitive Change Scores

Changes in cognitive performance from baseline to follow-up are presented in Table 1. We first present the analysis of the effect of iron treatment (60 mg and 80 mg combined) *versus* no treatment (placebo and control combined) on scores for each cognitive domain. Following this, scores on each of the individual measures that contribute to the domain scores are compared between treatment and no treatment groups. Finally, domain scores and the individual measures that contribute to the domain scores were analysed for ferritin improvers *versus* non-improvers, and haemoglobin improvers *versus* non-improvers. The results of the statistical analyses are presented in Table 2.

**Table 2:** Cognitive domain change scores for iron treatment and no treatment groups.

Cognitive change Sten score	Iron treatment groups (*n* = 13) (Mean ± SD)	No treatment groups (*n* = 12) (Mean ± SD)	*p* Value
Memory	0.67 ± 0.78	0.08 ± 1.76	0.210
Response speed	−0.27 ± 2.21	−0.46 ± 2.15	0.512
Impulsivity	0.62 ± 1.83	−0.88 ± 2.42	0.047 *
Attention	1.31 ± 2.80	−0.54 ± 2.36	0.085
Information Processing	1.00 ± 1.86	−0.21 ± 2.48	0.107
Executive Function	0.62 ± 2.48	0.58 ± 1.43	0.805
Emotion Identification	−0.50 ± 2.16	0.96 ± 1.67	0.105

Note: * Sig p < 0.05. Iron treatment groups = 60 mg and 80 mg iron, no treatment groups = iron-deficient placebo and iron-sufficient controls.

While statistically significant differences were not necessarily expected due to the small sample size, analysis of cognitive domain change scores (follow-up minus baseline) revealed that Impulsivity improved significantly more in the treatment (60 mg iron and 80 mg iron combined) than no treatment groups (placebo and controls combined) (*p* = 0.047). There were no other statistically significant differences in cognitive domain change scores between treatment and no treatment groups at the *p* < 0.05 level. At the *p* < 0.10 level, Attention change scores were larger for treatment than no treatment groups (*p* = 0.085).

Analysis of change scores for the individual measures that contribute to the domain scores found that the iron treatment groups had significantly higher recognition memory change scores than the placebo groups (*p* *=* *0*.029). There were no other statistically significant differences in cognitive change scores between the iron treatment and the no-treatment groups at the *p* < 0.05 level. At the *p* < 0.1 level, the iron treatment groups showed a greater improvement in reaction time on a sustained attention task (correctly pressing the same letter twice in a row) than the no-treatment groups (*p* = 0.064). For the Go/No-go task, reduction in total errors (*p* = 0.053) and omission errors (*p* = 0.083) was also greater for treatment than no treatment groups. In contrast, the results of an emotion recognition task showed that the placebo groups had a greater change score for correctly identifying fear faces than the iron treatment groups (*p* *=* 0.056), and a greater improvement in reaction time scores for that task than the iron treatment groups (*p* *=* 0.050).

##### 3.3.3.1. Analysis of Cognitive Scores in Ferritin Improvers and Non-Improvers

Cognitive domain change scores for Impulsivity and Attention were significantly greater for ferritin improvers than non-improvers (*p* = 0.004, *p* = 0.026, for Impulsivity and Attention, respectively). Change scores for Emotion Identification were significantly smaller for ferritin improvers than non-improvers (*p* = 0.022) (Table 3).

**Table 3:** Cognitive domain change scores for ferritin improvers and non-improvers.

Cognitive change Sten score	Ferritin improvers (*n* = 17)	Ferritin non-improvers (*n* = 8)	*p* Value
Memory	0.69 ± 0.95	−0.25 ± 1.87	0.071
Response Speed	0.15 ± 2.49	−0.06 ± 1.40	0.930
Impulsivity	0.74 ± 2.07	−1.88 ± 1.36	0.004 *
Attention	1.24 ± 2.49	−1.31 ± 2.43	0.026 *
Information Processing	0.62 ± 1.91	0.00 ± 2.88	0.334
Executive Function	0.74 ± 2.21	0.31 ± 1.56	0.681
Emotion Identification	−0.44 ± 2.05	1.56 ± 1.24	0.022 *

Note: * Sig *p* < 0.05. Ferritin improvers are those whose ferritin increase by more than the known biological day-to-day variation of 27%, non-improvers either had no change or a decline in ferritin.

Analysis of individual measures that contribute to the domain scores found that ferritin improvers had a greater improvement in recognition memory compared with non-improvers (*p* *=* 0.003). Change scores for a sustained attention task were also significantly greater for ferritin improvers than non-improvers (*p* = 0.048). For the Go/No-go task, ferritin improvers had a greater reduction in total errors (*p* = 0.005) and omission errors (*p* *=* 0.009) than non-improvers. Ferritin non-improvers had a greater improvement in reaction time to identify fear and sad faces than the ferritin improvers (*p* *=* 0.023, *p* *=* 0.023). There were no other statistically significant differences in cognitive change scores between ferritin improvers and non-improvers at the *p* < 0.05 level. At the *p* < 0.1 level, on the Go/No-go task, ferritin improvers had a greater improvement in reaction time variability (*p* *=* 0.081), and omission errors (*p* *=* 0.067) than non-improvers. Ferritin improvers also had greater improvement than non-improvers on digit span forwards (*p* = 0.069), and a greater reduction in total errors on a sustained attention task (*p* = 0.068).

##### 3.3.3.2. Analysis of Cognitive Scores in Haemoglobin Improvers and Non-Improvers

Analysis of cognitive domain change scores showed no significant differences between haemoglobin improvers and haemoglobin non-improvers (Table 4).

**Table 4:** Cognitive domain change scores for haemoglobin improvers and non-improvers.

Cognitive change Sten score	Haemoglobin improvers (*n* = 10)	Haemoglobin non-improvers (*n* = 15)	*p* Value
Memory	0.61 ± 1.50	0.23 ± 1.31	0.741
Response Speed	0.30 ± 1.75	−0.07 ± 2.46	0.521
Impulsivity	0.05 ± 2.23	−0.20 ± 2.29	0.739
Attention	0.75 ± 2.47	0.20 ± 2.92	0.802
Information Processing	1.35 ± 2.56	−0.20 ± 1.79	0.112
Executive Function	0.65 ± 2.12	0.57 ± 1.99	0.538
Emotion Identification	0.50 ± 1.43	0.00 ± 2.39	0.780

Note: Haemoglobin improvers are those whose haemoglobin increase by more than the known biological day-to-day variation of 4.4%, non-improvers either had no change or a decrease in haemoglobin.

Analyses of individual measures that contribute to the domain scores found that haemoglobin improvers had significantly greater improvement on digit span forwards (*p* *=* 0.034) and greater improvement in accuracy on a switching of attention task (*p* = 0.022) than non-improvers. There were no other statistically significant differences in cognitive change scores between haemoglobin improvers and non-improvers at the *p* < 0.05 level. At the *p* < 0.1 level, haemoglobin improvers had a greater reduction in errors on a verbal interference task, (*p* *=* 0.064) compared with non-improvers. Haemoglobin improvers also had a larger reduction in reaction time to identify happy faces than non-improvers (*p* = 0.067).

#### 3.4. Effect of Guessing Treatment Allocation on Cognitive Change

Within the treatment group, there were no significant differences in cognitive change scores between participants who correctly guessed that they were taking iron and those who did not (Memory *p* = 0.130, Response Speed *p* = 0.946, Impulsivity *p* = 1.00, Attention *p* = .462, Information Processing *p* = 1.00, Executive Function *p* = 0.382, Emotion Identification *p* = 0. 893). Within the no-treatment group, participants who incorrectly guessed they were taking iron had a significantly lower Attention change score than those who correctly guessed they were taking placebo (0.50 ± 1.87, −2.0 ± 2.34, *p* = 0.026). There were no other significant differences in cognitive change scores between participants who correctly guessed that they were taking placebo and those who did not (Memory *p* = 0.456, Response Speed *p* = 0.286, Impulsivity *p* = 0.683, Information Processing *p* = 0.935, Executive Function *p* = 0.459, Emotion Identification *p* = 0. 621).

#### 3.5. Required Sample Size for an Adequately Powered RCT

Power calculations were conducted to determine sample size for a future adequately powered RCT. Based on the difference in Memory scores between iron-deficient participants on iron treatment (60 mg and 80 mg) and those on placebo, 26 participants would be required in each iron treatment group for an adequately powered RCT. Based on scores in the Attention domain, a sample size of 84 iron deficient participants would be required in each treatment group for an adequately powered RCT.

## 4. Discussion

This is the first study to report on the use of the IntegNeuro battery of cognitive tests for assessing cognition in iron-deficient young women. IntegNeuro is a validated tool that is suitable for people aged 6–96 years [26]. It is available internationally, with versions in English, Spanish, Dutch and Hebrew [35]. The battery can be self-administered using a standard personal computer and touchscreen monitor. Scoring of test results is done centrally by the Brain Resource Company (San Francisco, CA, USA) and standardised against the International Brain database which contains data from more than 20,000 people [35]. IntegNeuro therefore offers the potential for researchers around the world to use a highly standardised and controlled method for cognitive research generally, but would allow some homogeneity in testing methods in the area of iron deficiency and cognition, which is currently lacking.

### 4.1. Suitability of IntegNeuro for Iron Deficiency Research

The primary aim of this pilot study was to examine the suitability of the IntegNeuro battery of tests for assessing Cognitive Function in iron-deficient and iron-sufficient young women. The IntegNeuro was simple to administer and well accepted by the population of young women included in the study. Three participants reported that the response time of the touchscreen was slow, but no other problems were reported. The process of uploading data to the Brain Resource Company for scoring and subsequent return of standardised data occurred without incident. Logistically, the IntegNeuro was found suitable for use in research in iron-deficient and iron-sufficient young women.

The suitability of the IntegNeuro battery to provide useful cognitive data in iron-deficient young women and trials of its treatment are less clear from this small pilot study. There were no differences detected in cognition scores for any domains for iron-deficient *versus* iron-sufficient young women at baseline. However, there were some statistically significant differences in cognitive domain change scores for ferritin improvers (irrespective of treatment group) compared with non-improvers, including the Attention and Impulsivity domains.

### 4.2. Required Sample Size for an Adequately Powered RCT

The secondary aim of this pilot study was to determine an appropriate sample size for an adequately powered RCT. Previous research has shown a relationship between iron deficiency and performance on Memory and Attention tasks [16,17,18,20], therefore these cognitive domains were used in the sample size calculations. The sample size used in this pilot study was insufficient to detect a statistically significant difference in cognitive function between groups at baseline for Memory and Attention and this should be considered when interpreting the results. More importantly, the results of the power analyses provided guidance regarding sample size for a future RCT.

### 4.3. Effect of Iron Deficiency on Cognition at Baseline and after Iron Treatment

This study found no statistically significant differences for any of the cognitive domains for iron-deficient *versus* iron-sufficient women at baseline. Participants in iron treatment groups had significantly higher change scores for the Impulsivity domain, and an individual task for Memory compared with the no-treatment groups.

While there has been limited research to determine the effect of iron deficiency on cognitive function in young women [23], of the studies that do exist, there is a great deal of heterogeneity in cognitive testing methods and specific sample populations (pregnant, overweight, receiving medical treatment) making comparisons difficult [1,10]. A recently conducted systematic review [23] reports on eight studies that included both iron-deficient and iron-sufficient participants at baseline [15,16,17,18,19,20,39,40]. Of these, four reported higher cognitive scores for iron-sufficient than iron-deficient participants at baseline, as well as improved scores after iron treatment [15,16,17,20]. Three studies reported no difference in cognition between iron-deficient participants compared with iron-sufficient controls at baseline [18,19,39]. These studies did show improvement in cognitive function in previously iron-deficient participants after iron treatment. One study showed no difference in levels of concentration between iron-deficient and iron-sufficient groups either at baseline or following iron treatment, which the authors attributed to small sample size (*n* = 375) [40].

Two of the studies included in the systematic review recruited only iron-deficient participants [21,22]. One of these studies reported an improvement in performance on cognitive tasks following iron treatment [21], and the other study showed no difference in cognitive function after iron treatment [22]. The latter study was limited by the use of haemoglobin as the only marker of iron status, with no real screening to determine cause of anaemia [22]. Differences in the effect of iron deficiency on cognitive function reported between studies may be due to the different cognitive tools used, as some used individual tests (such as digit span, digit symbol [18] and maze test [22], sustained attention [21]) and others used composite test batteries (such as the Cambridge Neuropsychological Test Automated Battery [24], the Cognitive Abilities Test [16]). Variations in sample size between the studies do not appear to be a contributor to the differences in results reported. The sample size in studies were varied (e.g., *n* = 152 [16], *n* = 53 [15], *n* = 222 [20], *n* = 76 [17], *n* = 24 [19], *n* = 95 [39], *n* = 38 [18], *n* = 375 [40], *n* = 38 [18]).

Blanton [24] has very recently reported improvements in the individual tasks of planning speed and spatial working memory strategy in previously iron-deficient women (*n* = 54). Prior to this, Murray-Kolb and Beard [16] performed a large (*n* = 149) well controlled study that showed improvements in the cognitive domains of attention, memory and learning in previously iron-deficient and iron-deficient anaemic young women after iron treatment [16]. Both Blanton and Murray-Kolb and Beard’s studies differed from the previous studies in the way in which they were analysed. Rather than examining the data by treatment group, or “intention to treat” they instead used analyses that compared those participants who had an improvement in iron status (ferritin and/or haemoglobin) with those who showed no improvement, irrespective of treatment. While this is not usual practice, it can be justified by the large variations in response to iron treatment for iron deficiency, which may be due to individual variations in iron losses and in gastrointestinal iron absorption. In fact, our serum ferritin data were not consistent with what might be expected across 60 mg, 80 mg and placebo treatment groups [37]. For example, some improvements were seen in the placebo group (not associated with inflammation as measured by alpha-1-glycoprotein) and some decreases were seen in the treatment groups [37]. Therefore it was decided to analyse the data in a similar manner to Blanton and Murray-Kolb and Beard and this resulted in the detection of some significant changes in domain and individual cognitive scores for ferritin improvers and individual scores in haemoglobin improvers.

Most participants in the iron treatment group correctly guessed their treatment allocation [37]. However, there was no statistical difference in participants’ ability to guess their treatment allocation between the treatment and placebo groups. As reported above, participants who guessed that they were taking iron, when they were in fact taking placebo, actually performed worse on the Attention task than those who correctly guessed that they were taking placebo.

Limitations of the current study include that participants were primarily university educated, which is not necessarily representative of reproductive aged females in the community. Another limitation is that strict instructions regarding the time of the day to take capsules were not provided because it was felt that an additional degree of burden regarding time restrictions may have reduced compliance. Further, this study did not control for the dietary intake prior to or during testing, menstrual cycle, exercise habits, the use of stimulants, sleep patterns or stress prior to testing. The time of the day for cognitive or blood testing were also not controlled, to accommodate for the busy schedules of the volunteers. Participants were asked to have their blood samples collected within the following 24–48 h after cognition testing. However, due to logistical barriers associated with participants’ availability and the Hunter Area Pathology availability, the duration of time between cognitive testing and blood sample collection varied between participants. Regarding compliance, efforts were made to remind participants to take capsules, some chose not to have text message reminders. However strengths were that the double blinded intervention and the use of validated assessment of cognitive function adhere to the Consolidated Standards of Reporting Trials statement [41].

## 5. Conclusions

IntegNeuro is an easy to administer tool for the assessment of cognition in young women. Some cognitive change scores were significantly higher for ferritin improvers (irrespective of treatment group) than non-improvers, and for women who had latent iron deficiency at baseline and were treated with iron supplements. Further research, using a larger sample of approximately 26–84 iron-deficient participants in each group, is required to determine the effectiveness of IntegNeuro in assessing the relationship between iron deficiency and cognitive function in this population.
